# Individualized genomics and the future of translational medicine

**DOI:** 10.1002/mgg3.11

**Published:** 2013-05-15

**Authors:** Maximilian Muenke


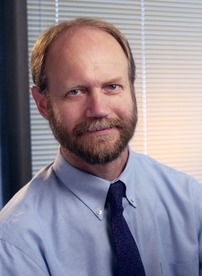


The age of genomic medicine is upon us. Sixty years have passed since Watson and Crick's discovery about the double-helical structure of DNA (Watson and Crick 1953). Ten years have passed since the Human Genome Project's (HGP) completion (International Human Genome Sequencing Consortium 2004). We now witness the translation of this basic science into direct patient care. With the cost of sequencing a human genome rapidly moving toward the $1000 mark, genomic evaluation in the everyday clinical setting is becoming a reality. Genomics has successfully been applied to many clinical areas, including rare disease diagnosis, pharmacogenetics, and a wide range of cancer diagnoses. Large databases and projects characterizing human variation such as the HapMap, the NHLBI Exome Variant Server, and the 1000 Genomes Project have been established and provide a touchstone for the interpretation of clinical genomic data.

Despite these and many other milestones, we are still at the dawn of the genomic age. Three billion pieces of the DNA puzzle have been placed on the table, but the puzzle is far from complete. The aim of the journal *Molecular Genetics & Genomic Medicine* (*MGGM*) is to not only participate in the assembly of the puzzle but also to help interpret the image as it is revealed, thereby bringing a new level of understanding to human, medical, and molecular genetics.

Rapidly evolving genomic technologies have given geneticists the tools to make efficient molecular diagnoses in diseases that had previously gone undiagnosed even after exhaustive genetic and clinical evaluations. At this moment, Online Mendelian Inheritance in Man (OMIM; http://www.omim.org) lists 4861 phenotypes with known molecular bases (Amberger et al. 2011; accessed March 2013). We see examples where a molecular diagnosis obtained from whole-exome sequencing (WES) has led to therapeutic benefit of the patient, such as the discovery of an inhibitor of apoptosis gene (*XIAP*) mutation that led to the successful allogeneic hematopoietic progenitor cell transplant in a boy with an unusually severe form of Crohn disease (Worthey et al. 2011). Investigations of rare disorders using WES have not only identified the respective causes but have led to biological insights that then impact on our knowledge about human disease architecture and our diagnostic capabilities. These successes have largely focused on single-gene mutations, each with large effect sizes. As whole-genome sequencing (WGS) becomes more affordable and its data analysis more robust, discoveries of this nature are envisioned to become commonplace.

Despite the anticipated success of WES and WGS in rare diseases, the common complex diseases, such as some types of cardiovascular disease and behavioral conditions, have not yet been translated effectively into broadly useful clinical information. Our initial high expectations that genome-wide association studies (GWAS) would provide clearer understanding of the inherited basis of common disorders have been tempered by modest effect sizes. Height is an example of a well-studied heritable complex trait (heritability of 0.8) with only a small amount of its variation explained by genetic variants. GWAS identified 54 loci in almost 63,000 people that only explained 5% of height variation (Visscher 2008). One notable example of a GWAS providing a greater than modest association is the increased risk of age-related macular degeneration with certain alleles of complement factor H (OR 2.27; 99% CI 2.10–2.45) (Sofat 2012). Regardless of the largely small effect sizes revealed by GWAS, the information derived from the HapMap Project allowed the power of hypothesis-free GWAS and the ability to map complex disease loci as a tool to provide new information regarding disease mechanisms, contributing to bench-to-bedside efforts.

GWAS have also exposed less characterized areas of the genome, as the majority of associated single-nucleotide polymorphic markers reside in non–protein-coding regions. Of the roughly three billion bases of the haploid genome, ∼1.5% code for proteins. This 1.5% has been the subject of intense scrutiny for disease relevance, while the remaining 98.5% of the genome formerly largely relegated as “junk”, now represents a new frontier. Projects such as the Encyclopedia of DNA Elements (ENCODE; http://encodeproject.org/encode) demonstrated that over 80% of our genome contains sequences that have a functional property (ENCODE 2012), pointing toward other areas of discovery including epistasis and epigenetics. Interestingly, epistasis may contribute to underestimating the effect of DNA sequence variants. As described conceptually by Eric Lander, instead of looking for more variants that comprise the numerator of heritability, we need to shrink the denominator by factoring in epistatic factors (Zuk et al. 2012). These new areas of research and discovery are adding to our understanding of the pathogenesis of complex phenotypes.

The exploration of our genome to discover the molecular basis of complex disease will require the study of diverse populations that differ in their minor allele frequencies (MAFs), as most genetic studies have been conducted primarily through the study of populations of European descent. By extending investigations into previously ignored groups, new insights into novel genomic variants will be found. An excellent example is the association of the gene *KCNQ1* which was found to be associated with type 2 diabetes in an Asian population, but was not significant in a larger European population based on a higher MAF in the Asian population, allowing for a larger power of detection (Unoki et al. 2008). Promoting research in other populations, projects such as the Human Heredity and Health in Africa (H3Africa) Initiative, which aims to facilitate genomics research in Africa, will provide a more complete understanding of the genomic underpinnings of disease in understudied populations (http://h3africa.org/).

The challenges to incorporating genomics into medical practice are many, and begin with our workforce. We simply do not have an appropriate workforce needed to provide the hours of counseling required to explain whole-exome/genome sequencing results to patients and research participants. With only 3026 Certified Genetic Counselors, this amounts to one counselor per 104,292 U.S. Americans (Brunham and Hayden 2012). The 1000 Genomes Project data suggest that each individual has 50–100 variants classified by the Human Gene Mutation Database (HGMD; http://www.hgmd.org) as causing inherited disorders (1000 Genomes Project Consortium et al. 2010). With this genetic burden, it will take time to adequately consent and provide results to patients, given the complex nature of the potential findings.

Other health-care professionals, especially primary care physicians, will be at the front lines to bring the genomics revolution to medical care, and they will need to be adequately trained. There is a known deficiency in genetic and genomic knowledge among primary care physicians, and filling this dearth will require creative solutions, both in terms of “educating the educators” as well as providing tools to help communicate genomic information to the public (Baars et al. 2005). Fortunately, the Centers for Disease Control and Prevention (CDC) has formed the Evaluation of Genomic Applications in Practice and Prevention (EGAPP), a working group that uses evidence-based methods to evaluate the validity and clinical utility of genomic testing. Examples of EGAPP projects include studies involving Factor V Leiden and venous thrombosis, type 2 diabetes, Lynch syndrome, and cardiovascular disease (http://www.egappreviews.org/). These types of robust evidence-based practice guidelines will be essential to both primary care physicians and geneticists.

Public perception and understanding of genomic medicine must be guided by early science education of our youth. Few members of the public possess the understanding of complex scientific concepts related to the genome. With the genomic landscape changing so quickly, properly educating our K-12 population will be imperative so that they can make decisions about their health care, participate in political decision making, and fill the ranks of our science and health-care professionals.

Part of the public's education must involve communicating realistic expectations for genomics. A *New York Times* article reported that 10 years after announcing the generation of a draft sequence of the human genome by the HGP, none of the promised benefits had been realized. This article astutely noted, for example, that a family history was more valuable than DNA variants for predicting cardiovascular disease in women (Wade 2010). In those intervening 10 years, there had actually been extraordinary progress in genomics; however, because somewhat unrealistic expectations were projected, some observers were disappointed.

As a significant impediment to the public's accurate understanding of genomics and genetics, the direct-to-consumer (DTC) genomic data industry continues to perpetuate false expectations. Certain DTC companies exhibit the tendency to fuel public misconceptions, stating that their clients will be able to manage risks and make decisions about their health with their genomic tests, when much of their testing is for complex diseases. As noted above, complex disease-associated variants are typically not clinically applicable at this time due to small effects on actual disease risk, most less than one percent. For these and other reasons, some DTC companies have changed their model and now require a health-care provider's referral before testing (https://www.pathway.com/). This type of test may be mainly ordered through primary care physicians who incompletely grasp the limited nature of the information provided by DTC; this reinforces the importance of genomic education and related evidence-based guidelines.

We now have numerous powerful tools such as WGS at our disposal. This allows the diagnosis of a symptomatic individual who in the past would have remained undiagnosed, enables targeted screening in asymptomatic individuals so as to intervene early in conditions such as Lynch syndrome, encourages therapeutic decisions based on pharmacogenomic information, and may better inform reproductive decisions. It is clear where we need to go: we must build an infrastructure of genetic and genomic professionals to channel the coming flood of WGS data, educate our public to make more informed decisions, create large-scale and open public databases and tools to support our clinicians, develop evidence-based guidelines, and promote research and continue to develop efficient functional tools directed at understanding the molecular basis of disease, with the ultimate goal of therapeutic benefit.

We are certain that the coming decades in genomics will be exciting. *MGGM* is committed to sharing these exciting developments with the research community. By publishing with open access, *MGGM* will share novel research and cutting-edge insights with the widest possible audience. Open access online publication is also in keeping with the growing expectation in the research community for freely available data about both rare Mendelian disorders and common diseases. Through the efforts of our outstanding editorial board, referees, and authors, a key goal of *MGGM* is to facilitate translational medical research from diagnostic laboratory to clinic, thereby helping to fulfill the great promise of genomic medicine.
